# The virtues of the virtual medical school interview

**DOI:** 10.1080/10872981.2021.1992820

**Published:** 2021-11-10

**Authors:** A. Peter Eveland, Lissett G. Prado, Sabrina R. Wilhelm, Stephanie Wong, Sanford H. Barsky

**Affiliations:** Department of Medical Education, Cancer Center and Institute for Personalized Medicine, California University of Science and Medicine

**Keywords:** Medical school admissions, medical school interviews, virtual interviews

## Abstract

The COVID-19 pandemic has mandated the use of virtual interactions in medical school. Although this falls mainly in the area of didactic instruction, of necessity, it has extended to the critical Admissions Process and the Medical School Interview itself. The California University of Science and Medicine (CUSM) with their flipped classroom approach had previously entered a virtual space of instruction even before COVID-19. Because CUSM was, in a sense, already committed to ‘virtual’ education, in the face of the COVID-19 pandemic, CUSM focused not on what it might lose but what it might gain and what their applicants to medical school might gain with the virtual format. The COVID-19 pandemic provided a unique opportunity to initially compare the Virtual Interview with the traditional On-Campus (In-Person) Interview during the hybrid 2020 year when the COVID-19 pandemic began. The Virtual Interview was patterned after the On-Campus Interview with some modifications. The same faculty conducted both interviews. A number of inherent advantages of the Virtual Interview surfaced to these faculty interviewers based on their subjective observations and conclusions. The overall interviewee satisfaction with the Virtual Interview was very positive based on their subjective observations and conclusions. The objective data from the Virtual Interviews compared to the On-Campus Interviews in the hybrid year resulted in a greater percentage of both offers of acceptance (p = .001) and matriculations (p = .001). In order to strengthen our initial observations, we expanded our study to include 2 pre-COVID-19 years (2018, 2019) of exclusively On-Campus interviews (n = 743) and 1 additional COVID-19 year (2021) of exclusively Virtual Interviews (n = 529). In this expanded study, interviewee demographics were not confounding and the Virtual Interview gave rise to overall greater interviewee satisfaction (p = .001), a trend to greater interviewer satisfaction and a greater percentage of both offers of acceptance (p = .047) and matriculations (p = .036).

## Introduction

Under the best of circumstances, medical school admissions is an arduous process [1,2]. Each year, medical schools across the country routinely receive thousands of applications for just a few hundred positions. Admissions committees have the daunting task of selecting, for their institution, those applicants who will best fulfill their mission. An inviolate cornerstone of the admission’s process has been the medical school interview that traditionally has always been conducted in person [3,4]. When COVID-19 entered our landscape, the pandemic dictated that allopathic medical schools transition to a virtual classroom approach for didactic sessions [5–8]. In addition, those medical schools who had yet to finish their admissions process found themselves scrambling to come up with an alternative means by which they could accurately and appropriately complete this year’s admissions cycle. The California University of Science and Medicine’s School of Medicine (CUSM) was one of those institutions. CUSM’s admissions committee had only 1 week to develop a viable means to conduct the remaining admissions day interviews, online, in a way that carried forward the intent and integrity of the previous on campus interview day process and in a way that was fair to both the past applicants who had been interviewed in person as well as the future applicants to be interviewed virtually.

CUSM with their flipped classroom, in a sense, had previously entered a virtual space of instruction even before COVID-19. Because CUSM was, in a sense, already committed to ‘virtual’ education, in the face of the COVID-19 pandemic, CUSM focused not on what it might lose but what it might gain and what their applicants to medical school might gain with the virtual format. The COVID-19 pandemic provided a unique opportunity to compare the Virtual Interview with the traditional On-Campus (In-Person) Interview.

In this report, we initially communicate our experiences with the Virtual Interview in the hybrid 2020 year when COVID-19 began, compare its strengths and weaknesses both in terms of interviewers’ perceptions and students’ evaluations and further comment on things that were not initially appreciated but were realized during the experience. Our overriding initial question, of course, was whether the Virtual Interview could be a viable and adequate surrogate for the On Campus (In-Person) Interview, a question that had been raised even before COVID-19 [9].

After studying the virtual interview in the hybrid 2020 year, we decided to expand our study to include the pre-COVID-19 years of 2018 and 2019 where all the interviews were conducted On-Campus (In-Person) and the most recent COVID-19 year of 2021 where all the interviews were conducted virtually. This expanded study included the required number of applicants and interviewees that, according to our power calculations, would be anticipated to yield meaningful and statistically significant results.

## Materials and methods

The study was initiated in 2020 after the COVID-19 pandemic struck and was originally limited to only On-Campus (In-Person) and Virtual Interviews conducted during the 2020 admissions year.

Each element of CUSM’s normal on-campus interview day agenda was reviewed with the goal of determining how each facet could be duplicated in a virtual environment. Only one item was not carried into the virtual interview day experience, a tour of our temporary medical education building. This segment was not offered as the class entering that fall would be located in our new forty-million-dollar education building and a virtual tour of that facility was not feasible at the time. This one feature, not withstanding, every other attempt was made to duplicate, as much was feasible, the features on the On-Campus Interview with the Virtual Interview.

The study was conducted under FERPA guidelines. All data had been collected as part of the routine admissions process. All subjects were de-identified. The present study was approved by CUSM’s IRB (HS-2020-04). The study was conducted blindly. But no blinded study has blinds on everyone. Certainly the interviewers and three of the authors of our paper (APE, SRW and LGP) were not blinded and knew the identity of the interviewees and the type of interview that was being conducted. These participants in the study served as ‘honest brokers’. But two of the authors of the study (SW, SHB) were completely blinded and were not made aware of any subject identifiers in their determination of power calculations or in the analysis of the data and their significance. Therefore from this perspective, the study was conducted blindly.

For the interviewees, the questionnaires, which were distributed and returned via E-mail, while not initially anonymous, were de-identified by the honest brokers and therefore remained anonymous to the other coauthors of the study and most importantly, to members of the Admissions Committee. Therefore, the responses of the interviewees were not linked to admission decisions. This was true both for the 2020 interviewees of the initial study as well as for the added 2018, 2019 and 2021 interviewees of the expanded study.

Observations of the interviewers were recorded during and after the interview process. Satisfaction levels of the interviewers who participated in either the On-Campus or the Virtual Interview were also compared. Comparisons of values between the groups were conducted using chi-square Tests of significance. An alpha value of ≤0.05 was considered significant. As a general rule only one interviewer interviewed one candidate at a time in both the On-Campus as well as Virtual Interview as part of the official faculty interview. However, in both the On-Campus as well as the virtual setting, faculty interviewers also conducted informal interviews in a conference room (In-Person) or virtually with multiple applicants as a means of answering questions about the school or answering or fielding group questions. But this was not part of the official formal interview and was only a very minor component of interview day.

Rates of acceptance and matriculation rates were compared initially between the On Campus *v* Virtual Interviewees of the hybrid 2020 year.

Following the hybrid 2020 admissions year, we expanded our study of acceptance and matriculation rates to include the pre-COVID-19 years of 2018 and 2019 where all the interviews were conducted in person and the most recent COVID-19 year of 2021 where all the interviews were conducted virtually.

The primary objectives of both our original study as well as our expanded study were twofold: 1) to compare the Virtual Interview with the traditional In-Person medical school interview and 2) in doing so, discover both the virtues as well as the shortcomings of the virtual interview. With these objectives in mind we approached the power calculations to ensure that sufficient power existed to detect significant differences or show that significant differences did not exist.

## Results

### Initial results from the 2020 hybrid year

The elements of the On-Campus (In-Person) interview are depicted (Supplement 1 – In Person Interview Schedule) that were also used for the Virtual Interview. The first step in the process was to send E-mail notifications to scheduled applicants regarding the Interview Day process moving it to an online, Virtual Interview Day format. The second step was to inform the interviewees of the different and additional preparatory steps that were required. The third step was providing applicants with the details of the actual Virtual Interview Day and the final step was a post-Virtual Interview Day follow-up. These steps were carried out in separate but sequential E mails. The details of all of these steps were sent to the interviewees at the appropriate times (Supplement 2 – Virtual Interview Schedule).

The same 15 faculty members participated in both the On-Campus as well as the Virtual Interviews. There were a total of 554 interviews for 130 slots. 477 interviews were conducted on campus and 77 interviews were conducted virtually. There were a total of 272 offers of acceptance, 227 made to the On-Campus interviewees and 45 made to the virtual interviewees. There was a total of 130 applicants who accepted the offers and matriculated, Of these, 99 matriculants had been interviewed on campus and 31 matriculants had been interviewed virtually. These data are summarized (Table 1).

**Table 1.** On campus *v* virtual interviews, offers of acceptance and matriculation, 2020 hybrid year

	Total Interviewees	On Campus Interviewees	Virtual Interviewees
N	554	477	77
Percentage	100%	86%	14%
Offers of Acceptance: N	272	227	45
Matriculants: N	130	99	31
Offers of Acceptance %	49%	48%*	58%*
Matriculants %	23%	21%*	40%*
Matriculant/Acceptance %	40%	44%*	49%*

Comparisons of values between the groups were conducted using Chi Square Tests of significance. An alpha value of ≤ 0.05 was considered significant.

*Significant at p = .001.

#### Subjective observations and conclusions of the interviewers

After conducting a number of interviews each interviewer made a number of observations that were recorded and pooled.

1. In the virtual format, interviewee and interviewer face each other on the screen with one on one interviews. This format has many advantages foremost of which it puts the interviewer and the interviewee on an equal footing. The interviewer is not sitting behind a large desk in a large professorial chair. But in the virtual format, from positioning considerations alone, interviewer and interviewee are equal.
2. In either situation where there are multiple interviewees with a single interviewer or multiple interviewers of a single interviewee, everyone faces each other on the screen and engages each other. This is vastly superior to interviewers and interviewees gazing across tables or shifting their eyes from different perspectives. The virtual interview fosters a sense of connection and trust. Trust grows when every face is equally visualized.
3. Both interviewer and interviewee are usually at home. This is especially important for the interviewee. Being at home makes the interviewee feel safe and secure and the interviewee is more apt to share his/her experiences openly and his/her reasons for wanting to become a physician. Being in a home environment would also be conducive to the interviewee feeling more comfortable discussing his/her personal history with the interviewer.
4. The virtual interview fosters both interviewer and interviewee to be nearly 100% present and engaged. This engagement fosters openness and honesty. However if during the interview the subject matter becomes emotional or sensitive, and the interviewee needs a break, the interviewer can allow the interviewee this break in the convenience and security of his/her home environment.
5. The virtual interview is both cost and time effective. It eliminates costly plane trips and hotel expenses. It eliminates travel time to and from the interview.
6. The virtual interview is conducive to questions emanating from both interviewer and interviewee and as such facilitates an exchange of ideas and creates a learning experience for both interviewer and interviewee.

#### Subjective observations and conclusions of the interviewees

The applicants who were interviewed virtually and matriculated overall had a very favorable impression of the process (Table 2).

**Table 2.** Virtual interview day questionnaire for hybrid year 2020

1. Overall, which of the following describes your impression of the CUSM Virtual Interview Day experience?	Answer Choices	Percentages	Responses
	Outstanding	45.45%	20
	Excellent	38.64%	17
	Good	18.18%	8
	Needs a lot of work	0.00%	0
2. Overall, based on my experience with the CUSM Virtual Interview Day, I would do it again.	Answer Choices	Percentages	Responses
	Yes	95.45%	42
	No	4.55%	2
3. Overall, the virtual nature of my one-on-one interview was as good as if I had experienced it in person.	Answer Choices	Percentages	Responses
	Strongly agree	25.00%	11
	Agree	50.00%	22
	Ambivalent	15.91%	7
	Disagree	6.82%	3
	Strongly disagree	2.27%	1
4. Overall, the virtual video presentations provided were helpful and informative.	Answer Choices	Percentages	Responses
	Yes	97.73%	43
	No	2.27%	1
5. My virtual lunch with current students was:	Answer Choices	Percentages	Responses
	Very satisfying	25.00%	11
	Satisfying	50.00%	22
	Somewhat satisfying	18.18%	8
	Not satisfying	4.55%	2
6. I completed my pre-interview assignments satisfactorily before participating in the Virtual Interview Day.	Answer Choices	Percentages	Responses
	Yes	97.73%	43
	No	2.27%	1
7. Were you glad to have had the opportunity to participate in the Virtual Interview Day experience.	Answer Choices	Percentages	Responses
	Yes	97.73%	43
	No	2.27%	1

#### Objective data from the virtual interviewees compared to the on campus interviewees

Only a minority of interviewees were virtual yet their offers of acceptance rate was greater than the On-Campus interviewees (p = .001). Even more striking, their matriculation rate was even greater (p = .001) (Table 1).

### Expanded results from the pre-COVID-19 in person interview 2018, 2019 years and the 2021 virtual interview year

#### Power calculations

Power calculations were determined with certain assumptions. Each power calculation relied on an alpha value (typically 0.05) and a calculation of the number of samples needed for a particular power, assuming a certain standard value, e.g. 80%. Comparing acceptance rates for Virtual *v* On-Campus interviews, we assumed an average medical school acceptance rate nationwide of approximately 7%.

**Figure 1.**
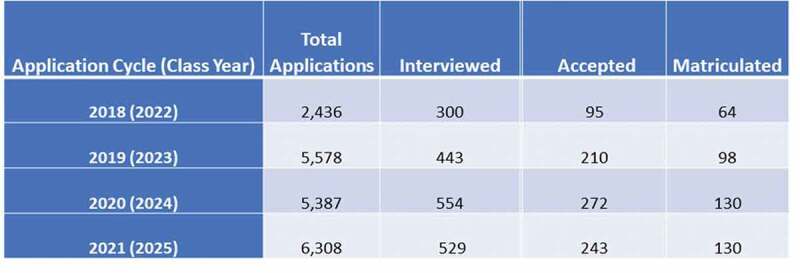
Expanded study. Expanded 4 year admissions data indicates the number of applications, interviewees, acceptances and matriculants. Power calculations (Supplement 3 – Figure 1 related power calculations), enabled detection of significant differences in many comparative studies

Based on these assumptions we derived a set of power calculations. We then determined the number of applicants, interviewees, offers of acceptance and matriculations for both the On-Campus as well as the Virtual interviewees in our expanded years 2018, 2019 and 2021 as well as our hybrid 2020 year (Figure 1). In our expanded study, we increased the overall absolute numbers of virtual interviews from 77 to 606 and the relative numbers of virtual interviews from a percentage of 14% to a percentage of 33% and tested whether our power calculations could detect differences of significance (Supplement 3 – Figure 1-related Power Calculations) and, in fact, significant differences were detected for most of the comparisons. The only exception to this was in our analysis of interviewer satisfaction that was limited to only the 15 faculty who conducted the interviews. With this limited number, only trends could be observed.

#### Lack of demographic confounding

Demographic breakdown on the basis of gender and ethnic background was distributed similarly in all admission years (Table 3) and therefore was not confounding in the Virtual *v* On-Campus interviewee analysis (p- = .447; p = .909).

**Table 3.** Demographics of expanded study

		Class of 2022		Class of 2023		Class of 2024		Class of 2025	
Demographics		N	%	N	%	N	%	N	%
**Sex**	Female	147	49%	195	44%	244	49%	259	49%
	Male	153	51%	248	56%	310	51%	270	51%
**Ethnicity**	Hispanic	54	18%	93	21%	100	18%	153	29%
	Asian	96	32%	182	41%	283	51%	180	34%
	Caucasian	129	43%	133	30%	122	22%	101	19%
	African American	3	1%	9	2%	11	2%	5	1%
	Other	18	6%	26	6%	38	7%	90	17%

#### *Virtual* v *On-Campus interviewee satisfaction*

**Figure 2.**
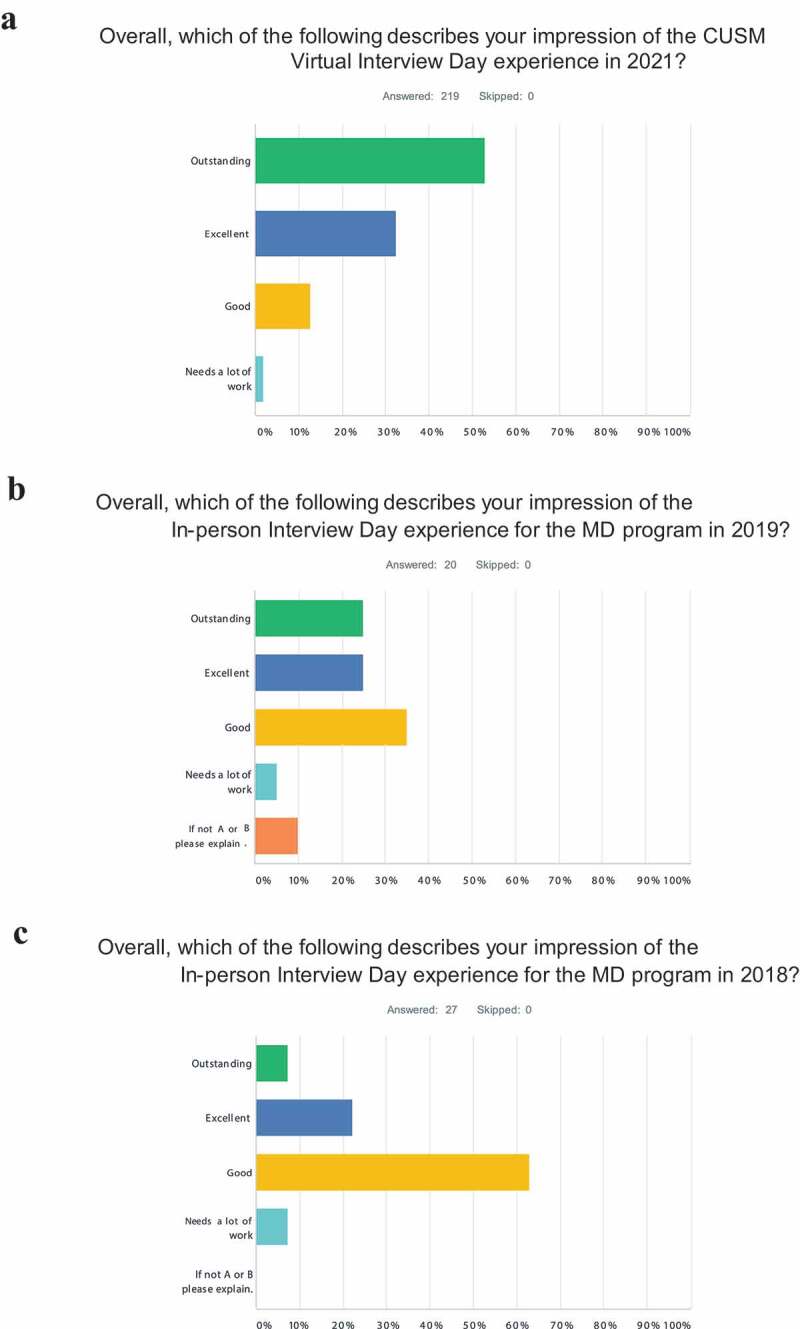
Satisfaction surveys of interviewees. Satisfaction of virtual interviewees (a) from application cycle (class year): 2021 (2025) is compared to On-Campus Interviewees (b) from 2019 (2023) and On-Campus interviewees (c) from 2018 (2022). The complete interviewee questionnaire is depicted (Supplement 4 – interviewee questionnaire)

With our now expanded data sets, we have conducted another interviewee survey of both types of interviews and categorized and compared their subjective responses in much more detail. Both virtual interviewees as well as On-Campus interviewees were generally satisfied with their respective interviews. However, the 2021 virtual interviewees uniformly responded to their questionnaires with higher levels of satisfaction than the 2018 and 2019 On-Campus interviewees (p = .001) (Figure 2). Furthermore this generally higher level of satisfaction was reflected in multiple other questions within their respective questionnaires (Supplement 4 – Interviewee Questionnaire). The questionnaires were conducted via E mail but the honest brokers (SRW, LGP) who received and analyzed the questionnaires then de-identified the interviewees. These honest brokers did not serve on the Admissions Committee and had no role in the Admissions process. Furthermore, the interviewees’ satisfaction scores were not linked to the Admissions process and the interviewees were so informed.

#### *Virtual* v *On-Campus interviewer satisfaction*

**Figure 3.**
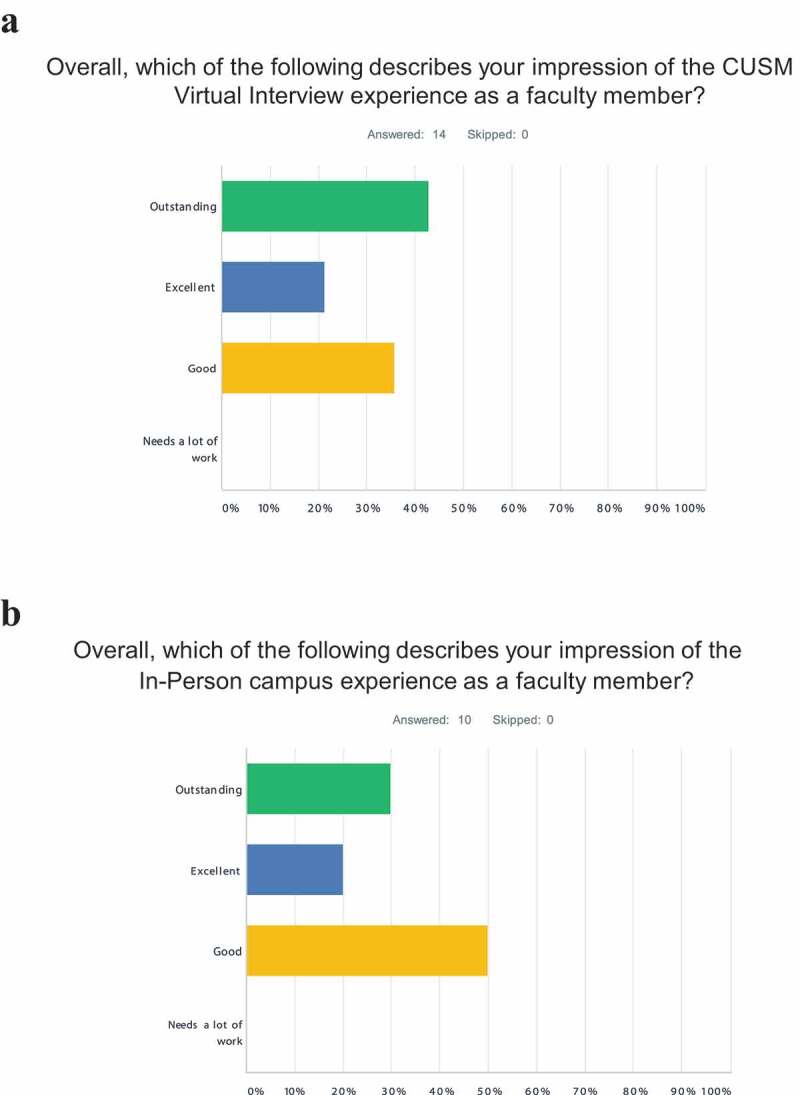
Satisfaction surveys of interviewers. Satisfaction of virtual interviewers (a) from application cycle (class year): 2021 (2025) is compared to On-Campus interviewers (b) from both 2019 (2023) and 2018 (2022). The complete interviewer questionnaire is depicted (Supplement 5 – interviewer questionnaire)

With our now expanded data sets, we have conducted another interviewer survey of the faculty involved with both types of interviews and categorized and compared their subjective responses in much more detail. Both virtual Interviewers as well as On-Campus interviewers were generally satisfied with their respective interviews. However, the 2021 virtual interviewers uniformly responded in their questionnaires with higher levels of satisfaction than the 2018 and 2019 On-Campus interviewers (Figure 3). Furthermore, this generally higher level of satisfaction was reflected in multiple other questions within their respective questionnaires (Supplement 5 – Interviewer Questionnaire). Due to the limited number of interviewer faculty, these observations could only be considered as trends.

#### *Virtual* v *On-Campus offers of acceptance and matriculation efficiencies*

The initial observations of greater offers of acceptance (p = .001) and matriculation (p- = .001) which was observed in the hybrid 2020 year was also observed when the virtual *v* On-Campus interviewees were combined in the expanded study involving years 2018–2021 (Table 4). The overall virtual offer of acceptance efficiency was 48% compared to the On-Campus offer of acceptance efficiency of 44% (p = .047). The overall virtual matriculation efficiency was 27% compared to the On-Campus matriculation efficiency of 21% (p = .036). Although the virtual matriculation/acceptance ratio was 56% compared to the On-Campus person matriculation/acceptance ratio of 49%, the differences only approached significance (p = .08).

**Table 4.** Hybrid year and expanded study comparisons

		Total Interviewees	On Campus Interviewees	Virtual Interviewees
2018, 2019	N	743	743	0
	Percentage	100%	100%	0%
	Offers of Acceptance: N	305	305	0
	Matriculants: N	162	162	0
	Offers of Acceptance %	41%	41%	0%
	Matriculants %	22%	22%	0%
	Matriculant/Acceptance %	53%	53%	0%
2020	N	554	477	77
	Percentage	100%	86%	14%
	Offers of Acceptance: N	272	227	45
	Matriculants: N	130	99	31
	Offers of Acceptance %	49%	48%*	58%*
	Matriculants %	23%	21%*	40%*
	Matriculant/Acceptance %	40%	44%*	49%*
2021	N	529	0	529
	Percentage	100%	0%	100%
	Offers of Acceptance: N	243	0	243
	Matriculants: N	130	0	130
	Offers of Acceptance %	46%	0%	46%
	Matriculants %	25%	0%	25%
	Matriculant/Acceptance %	53%	0%	53%
Overall	N	1826	1220	606
	Percentage	100%	67%	33%
	Offers of Acceptance: N	820	532	288
	Matriculants: N	422	261	161
	Offers of Acceptance %	45%	44%**	48%**
	Matriculants %	23%	21%***	27%***
	Matriculant/Acceptance %	51%	49%****	56%****

Comparisons of values between the groups were conducted using Chi Square Tests of significance. An alpha value of ≤ 0.05 was considered significant.

*Significant at p = .001.

**Significant at p = .047.

***Significant at p = .036.

****Approaching Significance at p = .08.

## Discussion

The COVID-19 pandemic has undoubtedly catalyzed the virtual approach to medical education including the interview process [10]. Our study was initially confined to the hybrid 2020 year and the relatively small numbers of interviewees limited the significance that could be assigned to our initial conclusions but even with these limitations, the potential virtues of the virtual interview in terms of satisfaction of both interviewee as well as interviewer as well as the efficiency of the process in terms of generation of offers of acceptance as well as matriculations surfaced.

Expanding the sample size of our study to include four full admissions years achieved sufficient power to detect a significant result (or reject the null hypothesis) when a true difference did exist. Furthermore, in our expanded study, we increased the overall absolute numbers of virtual interviews from 77 to 606 and the relative numbers of virtual interviews from a percentage of 14% to a percentage of 33%. Since this study was a comparative study, we felt that we needed a reasonable balance of virtual with traditional interviews and believe we achieved it. However, since it is predicted that virtual medical school admission interviews will continue in 2022 due to the persistence of COVID-19, a larger number of virtual interviews will be available shortly and this will allow addilitional comparative studies such as studies of academic performance metrics in those matriculants interviewed virtually with those matriculants interviewed traditionally.

In our expanded study, interviewee demographics were not confounding and the virtual interview gave rise to overall greater interviewee satisfaction (p = .001), a trend to greater interviewer satisfaction and a greater percentage of both offers of acceptance (p = .047) and matriculations (p = .049). Our expanded study validated and strengthened the conclusions of our initial study. Still both studies suffer from both a type of historical comparison as well as the subjectivity bias of both interviewees as well as interviewers.

With respect to the objective data from the Virtual Interviews compared to the On-Campus Interviews, we feel that many of the observations made are valid. The significantly higher rate of offers of acceptance and matriculation rate of the Virtual Applicants compared to the On-Campus Applicants is intriguing in its own right. Although there are several potential reasons for these findings, including the influence of the COVID-19 pandemic in stimulating offers of acceptance or matriculations or the belated nature of the Virtual Interview process in influencing offers of acceptance or matriculations late in the hybrid 2020 academic year and in the following 2021 year, it is attractive to consider the possibility that the virtues of the Virtual Interview per se facilitated both the institution’s decision-making process as well as the candidate’s confidence in matriculating. Review of the timing of offers of acceptance or matriculation decisions from the previous years’ class that were interviewed exclusively On-campus revealed that there was no belated bias in the timing of either the offer of acceptance or matriculations. Furthermore, our expanded data set involving not just one but 4 years also confirmed this. The findings of this study suggest the feasibility of at least using the Virtual Interview as a screening tool once the COVID-19 pandemic begins to subside to determine the strongest of the medical school applicants and then invite those candidates likely to be offered acceptances to visit the campus.

Nearly all the medical schools that we have contacted within the USA, because of the ongoing threat of COVID-19 especially in the context of travel, will be proceeding with virtual medical school admission interviews in 2022. Whether this trend persists after the pandemic becomes endemic remains to be seen but in any case the number of Virtual Interviews throughout the USA will definitely substantially increase in the short term and maybe in the long term as well. This will make the Virtual Interview even more relevant in our time.

In this initial study, we raise the hypothesis that the Virtual Interview, though still in its infancy, may yield many benefits and advantages still not recognized. It would be important to eventually conduct a prospective randomized study comparing the Virtual Interview with the On-Campus Interview. Only in this way can we validate the real virtues of the Virtual Interview.

## Supplementary Material

Supplemental Material

## Data Availability

All raw data, in de-identified format, is available to any interested party upon request. https://cusm.org/school-of-medicine/faculty/profiles/sanford-barsky.php

## Author titles

**A Pete Eveland** is Senior Associate Dean of Student Affairs and Admissions. **Lissett G. Prado** is Executive Assistant to the Associate Dean of Students. **Sabrina R. Wilhelm** is Director of Academic Skills and Career Advising. **Stephanie Wong** is a third year medical school student. **Sanford H. Barsky** is Executive Director of the Cancer Center and Institute for Personalized Medicine. All authors are from the California University of Science and Medicine.
