# Supplementary material for: The virtues of the virtual medical school interview
Source: Med Educ Online. 2021 Nov 10;26(1):1992820. doi: 10.1080/10872981.2021.1992820 (PMC8592618; doi:10.1080/10872981.2021.1992820)
Supplement: Supplemental Material [file ZMEO_A_1992820_SM8437.zip › Supplementary files/Supplement 2.docx]

**Supplement 2 - Virtual Interview Schedule**

As circumstances evolve, related to COVID-19, the California University of Science and Medicine School of Medicine (CUSM-SOM) has as our highest priority, protecting the safety and well-being of our applicants, students, staff, and faculty.

In order to minimize risk to our applicants, as well as to those with whom they might interact, we have determined to move all activities associated with interview day online. **Do not come to campus.**

We have transitioned to working remotely and have created an online interview process. At this time, all interview days will be rescheduled. We will be communicating with you as soon as we have all of the information and additional details.

Host/Moderator – Coordinated by Admissions Team Members

1. One week prior to scheduled Virtual Interview Day: An email was sent to applicants, which included the following:
   1. Request to RSVP
   2. The name of their host/moderator
   3. Virtual Interview Day Agenda
   4. Hotlinks to our video presentations, PowerPoint presentations, and handouts. (Sections IV & V)
2. Monday - prior to Interview Day on Friday: Assign and send invitation to faculty and applicants of their assigned interview date and time via MS Teams meeting. Also, hold a one- hour training session with faculty via MS Teams to view the process.
3. Wednesday – prior to Interview Day: Send an invite to our Student Admissions Ambassadors and applicants for a one-hour Q & A session. The host/moderators attend by audio only to monitor the session. The FAQ session is for both morning and afternoon applicants.
   1. Note: Student Admissions Ambassadors are students who were picked by the Office of Admissions to have lunch with applicants during the on-campus interview day. This transitioned into the one-hour Q&A session noted above.
4. Thursday prior to Interview Day: Host/Moderator (H/M) sends a “motivation message” to the applicants.
   1. Please note: The H/M is a member of the Admissions and Student Affairs staff who serves in various support capacities related to the Virtual Interview Day including:
5. Interview Day
6. The Host/Moderator (H/M) ensures that the applicant is logged into the MS Teams video conference 20 minutes prior to their scheduled appointment. Next, the H/M verifies that the faculty interviewers are logged into the conference five minutes prior to their interview appointment.
7. Post Interview Day

1. H/M send a “wrap up” email, which contains information on how to view faculty bios, university catalog, and student life information on our website.
2. Neo Assessment - Proctored Exam: The final step to the online interview process occurred three days after their one-on-one interview.
3. An email invitation was sent to the candidates by two members of the admissions staff with Ring Central instructions for participants to complete the NEO online.
   1. The NEO Five-Factor Personality Inventory provides reliable and valid information on the candidate from the big five domains of personality and six subsets under each domain. This effectively helps to accelerate our understanding of the candidate including the important data on personality characteristics of altruism, dutifulness, and conscientious. If the applicant is accepted and matriculates, the information obtained from the NEO is also used to help with placement into college groups with their peers.
   2. Invitations to complete the NEO were sent at least 3 days in advance. Reminder emails to the candidates to accept the meeting were sent and followed up with a final email reminder the day prior to the NEO appointment.
   3. Candidates received the NEO invitation link just prior to their appointment to that the inventory. At the appointed time, CUSM’s proctors went on line to greet the candidates and provide them with verbal instructions.
   4. Candidates were required to complete the inventory with a limit of 45 minutes. As candidates finished they were instructed to wave goodbye at the proctor, which signed their completion and electronic submission of the NEO.
