# Supplementary material for: The virtues of the virtual medical school interview
Source: Med Educ Online. 2021 Nov 10;26(1):1992820. doi: 10.1080/10872981.2021.1992820 (PMC8592618; doi:10.1080/10872981.2021.1992820)
Supplement: Supplemental Material [file ZMEO_A_1992820_SM8437.zip › Supplementary files/Supplement_3.docx]

| **Acceptance rate power calculation** |  |  | **Matriculation rate power calculation** |  |  | **Average satisfaction score** |  |
| --- | --- | --- | --- | --- | --- | --- | --- |
| alpha | 0.05 |  | alpha | 0.05 |  | alpha | 0.05 |
| power | 80 |  | power | 80 |  | power | 80 |
| in person acceptance rate | 3.8 |  | in person matriculation rate | 53% |  | in person avg satisf | 3.5 |
| virtual acceptance rate | 4.8 |  | virtual matriculation rate | 67% |  | virtual avg satisf | 3.75 |
| enrollment ratio | 0.79 |  | enrollment ratio | 0.63 |  | enrollment ratio | 1 |
| In person applicants needed | 7279 |  | in person offers of acceptance needed | 248 |  | in person interviewees needed | 16 |
| Virtual applicants needed | 5750 |  | virtual offers of acceptance needed | 156 |  | virtual interviewees needed | 16 |

**Supplement 3 – Figure 1 related Power Calculations**
