# Supplementary material for: The virtues of the virtual medical school interview
Source: Med Educ Online. 2021 Nov 10;26(1):1992820. doi: 10.1080/10872981.2021.1992820 (PMC8592618; doi:10.1080/10872981.2021.1992820)
Supplement: Supplemental Material [file ZMEO_A_1992820_SM8437.zip › Supplementary files/Supplement_4.docx]

**Supplement 4 – Interviewee Questionnaire**

Q1 Overall, which of the following describes your impression of the CUSM Virtual Interview Day experience in 2021?

Answered: 219 Skipped: 0

Outstanding

Excellent

Good

Needs a lot of work

0% 10% 20% 30% 40% 50% 60% 70% 80% 90% 100%

**ANSWER CHOICES RESPONSES**

Outstanding 52.97% 116

Excellent 32.42% 71

Good 12.79% 28

Needs a lot of work

1.83% 4

TOTAL 219

Q2 Overall, based on my experience with the CUSM Virtual Interview Day,

I would do it again.

Answered: 219 Skipped: 0

Yes

|  |  |  |  |  |  |  |  |  |  | |
| --- | --- | --- | --- | --- | --- | --- | --- | --- | --- | --- |
|  | | | | | | | | | |  |
| e  . |  |  |  |  |  |  |  |  |  | |

No

If no pleas explain

0% 10% 20% 30% 40% 50% 60% 70% 80% 90% 100%

**ANSWER CHOICES RESPONSES**

Yes 97.72% 214

No 0.46% 1

If no please explain.

1.83% 4

TOTAL 219

Q3 Overall, I felt the virtual nature of my one-on-one interview with faculty

was as good as if I had experienced the interview in person.

Answered: 219 Skipped: 0

A gree

Dis agree

If disagree , please explain .

0% 10% 20% 30% 40% 50% 60% 70% 80% 90% 100%

**ANSWER CHOICES RESPONSES**

Agree 86.76% 190

Disagree 2.74% 6

If disagree, please explain.

10.50% 23

TOTAL 219

Q4 The instructions I received to participate in the Virtual Interview Day provided me with the information needed to fully participate in the Virtual Interview Day event.

Answered: 219 Skipped: 0

Yes

No

If no, please explain.

0% 10% 20% 30% 40% 50% 60% 70% 80% 90% 100%

**ANSWER CHOICES RESPONSES**

Yes 95.43% 209

No 0.46% 1

If no, please explain.

4.11% 9

TOTAL 219

Q5 I did not experience any issues with the quality of my internet connection in preparation for or during the Virtual Interview Day experience.

Answered: 219 Skipped: 0

Yes

No

0% 10% 20% 30% 40% 50% 60% 70% 80% 90% 100%

**ANSWER CHOICES RESPONSES**

Yes 86.30% 189

No 13.70% 30

TOTAL 219

Q8 Overall, the virtual video presentations provided were helpful and informative.

Answered: 219 Skipped: 0

Yes

|  |  |  |  |  |  |  |  |  |  | |
| --- | --- | --- | --- | --- | --- | --- | --- | --- | --- | --- |
|  | | | | | | | | | |  |
|  |  |  |  |  |  |  |  |  |  | |

No

0% 10% 20% 30% 40% 50% 60% 70% 80% 90% 100%

**ANSWER CHOICES RESPONSES**

Yes 98.17% 215

No 1.83% 4

TOTAL 219

Q9 Overall, how satisfied were you with taking the NEO in an online and proctored environment?

Answered: 219 Skipped: 0

Very satisﬁed

Satisﬁed

S omewhat s atis ﬁed

Not satisﬁed.

If not

s atis ﬁed,...

0% 10% 20% 30% 40% 50% 60% 70% 80% 90% 100%

**ANSWER CHOICES RESPONSES**

Very satisfied 54.79% 120

Satisfied 36.07% 79

Somewhat satisfied 7.31% 16

Not satisfied. 0.46% 1

If not satisfied, please explain.

1.37% 3

TOTAL 219

Q10 My virtual lunch with current students was:

Answered: 219 Skipped: 0

Very satisfying

Satisfying

S omewhat s atis fying

Not satisfying

If not A or B

please explain.

0% 10% 20% 30% 40% 50% 60% 70% 80% 90% 100%

**ANSWER CHOICES RESPONSES**

Very satisfying 41.55% 91

Satisfying 44.75% 98

Somewhat satisfying 4.57% 10

Not satisfying 1.37% 3

If not A or B please explain.

7.76% 17

TOTAL 219

Q12 I completed the “Things to Do” assignments before participating in the

Virtual Interview Day activities.

Answered: 219 Skipped: 0

Yes

|  |  |  |  |  |  |  |  |  |  | |
| --- | --- | --- | --- | --- | --- | --- | --- | --- | --- | --- |
|  | | | | | | | | | |  |
|  |  |  |  |  |  |  |  |  |  | |

No

0% 10% 20% 30% 40% 50% 60% 70% 80% 90% 100%

**ANSWER CHOICES RESPONSES**

Yes 99.09% 217

No 0.91% 2

TOTAL 219

Q13 Approximately, how many miles do you normally live from our

campus?

Answered: 219 Skipped: 0

Over 100

75-100

50-75

Less than 50

0% 10% 20% 30% 40% 50% 60% 70% 80% 90% 100%

**ANSWER CHOICES RESPONSES**

Over 100 25.11% 55

75-100 9.13% 20

50-75 18.26% 40

Less than 50

47.49% 104

TOTAL 219

Q14 Were you glad to have had the opportunity to participate in the Virtual

Interview Day experience.

Answered: 219 Skipped: 0

Yes

|  |  |  |  |  |  |  |  |  |  |
| --- | --- | --- | --- | --- | --- | --- | --- | --- | --- |
|  | | | | | | | | | |
|  |  |  |  |  |  |  |  |  |  |

No

0% 10% 20% 30% 40% 50% 60% 70% 80% 90% 100%

**ANSWER CHOICES RESPONSES**

Yes 99.54% 218

No 0.46% 1

TOTAL 219

Q1 Overall, which of the following describes your impression of the

In-person Interview Day experience for the MD program in 2019?

Answered: 20 Skipped: 0

Outstanding

Excellent

Good

Needs a lot of work

If not A or B

please explain .

0% 10% 20% 30% 40% 50% 60% 70% 80% 90% 100%

**ANSWER CHOICES RESPONSES**

Outstanding 25.00% 5

Excellent 25.00% 5

Good 35.00% 7

Needs a lot of work 5.00% 1

If not A or B please explain.

10.00% 2

TOTAL 20

Q2 Overall, based on my experience with the CUSM-SOM in-person

Interview Day, I would do it again.

Answered: 20 Skipped: 0

Yes

No

If no please explain.

0% 10% 20% 30% 40% 50% 60% 70% 80% 90% 100%

**ANSWER CHOICES RESPONSES**

Yes 85.00% 17

No 5.00% 1

If no please explain.

10.00% 2

TOTAL 20

Q3 Overall, I felt the in-person nature of my one-on-one interview with

faculty was good.

Answered: 20 Skipped: 0

A gree

Dis agree

If disagree , please explain .

0% 10% 20% 30% 40% 50% 60% 70% 80% 90% 100%

**ANSWER CHOICES RESPONSES**

Agree 85.00% 17

Disagree 10.00% 2

If disagree, please explain.

5.00% 1

TOTAL 20

Q4 The instructions I received to participate in the Interview Day provided me with the information needed to fully participate in the event.

Answered: 20 Skipped: 0

Yes

No

If no, please explain.

0% 10% 20% 30% 40% 50% 60% 70% 80% 90% 100%

**ANSWER CHOICES RESPONSES**

Yes 85.00% 17

No 5.00% 1

If no, please explain.

10.00% 2

TOTAL 20

Q5 I did not experience any issues with the quality of the communication received for the preparation for or during the Interview Day experience.

Answered: 20 Skipped: 0

Yes

No

0% 10% 20% 30% 40% 50% 60% 70% 80% 90% 100%

**ANSWER CHOICES RESPONSES**

Yes 85.00% 17

No 15.00% 3

TOTAL 20

Q6 Which of the following applies to you?

Answered: 20 Skipped: 0

I have been accepted at...

Rejected

Placed on the

Alternate List

Don’t know

0% 10% 20% 30% 40% 50% 60% 70% 80% 90% 100%

**ANSWER CHOICES RESPONSES**

I have been accepted at CUSM 95.00% 19

Rejected 0.00% 0

Placed on the Alternate List 5.00% 1

Don’t know

0.00% 0

TOTAL 20

Q8 Overall, the in-person presentations provided were helpful and informative.

Answered: 20 Skipped: 0

Yes

No

0% 10% 20% 30% 40% 50% 60% 70% 80% 90% 100%

**ANSWER CHOICES RESPONSES**

Yes 95.00% 19

No 5.00% 1

TOTAL 20

Q9 Overall, how satisfied were you with taking the NEO in a proctored environment?

Answered: 20 Skipped: 0

Very satisﬁed

S atis ﬁed

S omewhat s atis ﬁed

Not satisﬁed.

If not

s atis ﬁed,...

0% 10% 20% 30% 40% 50% 60% 70% 80% 90% 100%

**ANSWER CHOICES RESPONSES**

Very satisfied 30.00% 6

Satisfied 35.00% 7

Somewhat satisfied 20.00% 4

Not satisfied. 10.00% 2

If not satisfied, please explain.

5.00% 1

TOTAL 20

Q10 My lunch with current students was:

Answered: 20 Skipped: 0

Very satisfying

Satisfying

S omewhat s atis fying

Not satisfying

If not A or B

please explain .

0% 10% 20% 30% 40% 50% 60% 70% 80% 90% 100%

**ANSWER CHOICES RESPONSES**

Very satisfying 45.00% 9

Satisfying 25.00% 5

Somewhat satisfying 10.00% 2

Not satisfying 0.00% 0

If not A or B please explain.

20.00% 4

TOTAL 20

Q12 I completed the “Things to Do” assignments before participating in the

Interview Day activities.

Answered: 20 Skipped: 0

Yes

No

0% 10% 20% 30% 40% 50% 60% 70% 80% 90% 100%

**ANSWER CHOICES RESPONSES**

Yes 75.00% 15

No 25.00% 5

TOTAL 20

Q13 Approximately, how many miles do you normally live from our campus?

Answered: 20 Skipped: 0

Over 100

75-100

50-75

Less than 50

0% 10% 20% 30% 40% 50% 60% 70% 80% 90% 100%

**ANSWER CHOICES RESPONSES**

Over 100 40.00% 8

75-100 10.00% 2

50-75 35.00% 7

Less than 50

15.00% 3

TOTAL 20

Q14 Were you glad to have had the opportunity to participate in the

Interview Day experience ?

Answered: 20 Skipped: 0

Yes

No

0% 10% 20% 30% 40% 50% 60% 70% 80% 90% 100%

**ANSWER CHOICES RESPONSES**

Yes 95.00% 19

No 5.00% 1

TOTAL 20

Q1 Overall, which of the following describes your impression of the

In-person Interview Day experience for the MD program in 2018?

Answered: 27 Skipped: 0

Outstanding

Excellent

Good

Needs a lot of work

If not A or B

pleas e explain.

0% 10% 20% 30% 40% 50% 60% 70% 80% 90% 100%

**ANSWER CHOICES RESPONSES**

Outstanding 7.41% 2

Excellent 22.22% 6

Good 62.96% 17

Needs a lot of work 7.41% 2

If not A or B please explain.

0.00% 0

TOTAL 27

Q2 Overall, based on my experience with the In-person Interview

Day, I would do it again.

Answered: 27 Skipped: 0

Yes

No

If no pleas e explain.

0% 10% 20% 30% 40% 50% 60% 70% 80% 90% 100%

**ANSWER CHOICES RESPONSES**

Yes 92.59% 25

No 7.41% 2

If no please explain.

0.00% 0

TOTAL 27

Q3 Overall, I felt the in-person nature of my one-on-one interview with faculty was good.

Answered: 27 Skipped: 0

A gree

Dis agree

If dis agree, pleas e explain.

0% 10% 20% 30% 40% 50% 60% 70% 80% 90% 100%

**ANSWER CHOICES RESPONSES**

Agree 85.19% 23

Disagree 11.11% 3

If disagree, please explain.

3.70% 1

TOTAL 27

Q4 The instructions I received to participate in the Interview Day provided me with the information needed to fully participate in the event.

Answered: 27 Skipped: 0

Yes

No

If no, pleas e explain.

0% 10% 20% 30% 40% 50% 60% 70% 80% 90% 100%

**ANSWER CHOICES RESPONSES**

Yes 88.89% 24

No 7.41% 2

If no, please explain.

3.70% 1

TOTAL 27

Q5 I did not experience any issues with the quality of the communication received for the preparation for or during the Interview Day experience.

Answered: 27 Skipped: 0

Yes

No

0% 10% 20% 30% 40% 50% 60% 70% 80% 90% 100%

**ANSWER CHOICES RESPONSES**

Yes 92.59% 25

No 7.41% 2

TOTAL 27

Q8 Overall, the in-person presentations provided were helpful and informative.

Answered: 27 Skipped: 0

Yes

No

0% 10% 20% 30% 40% 50% 60% 70% 80% 90% 100%

**ANSWER CHOICES RESPONSES**

Yes 88.89% 24

No 11.11% 3

TOTAL 27

Q9 Overall, how satisfied were you with taking the NEO in a proctored environment?

Answered: 27 Skipped: 0

V ery s atis ﬁed

S atis ﬁed

S omewhat s atis ﬁed

Not satisﬁed.

If not s atis ﬁed,...

0% 10% 20% 30% 40% 50% 60% 70% 80% 90% 100%

**ANSWER CHOICES RESPONSES**

Very satisfied 18.52% 5

Satisfied 48.15% 13

Somewhat satisfied 18.52% 5

Not satisfied. 7.41% 2

If not satisfied, please explain.

7.41% 2

TOTAL 27

Q10 My lunch with current students was:

Answered: 27 Skipped: 0

V ery s atis fying

Satisfying

S omewhat s atis fying

Not s atis fying

If not A or B

pleas e explain.

0% 10% 20% 30% 40% 50% 60% 70% 80% 90% 100%

**ANSWER CHOICES RESPONSES**

Very satisfying 14.81% 4

Satisfying 33.33% 9

Somewhat satisfying 3.70% 1

Not satisfying 3.70% 1

If not A or B please explain.

44.44% 12

TOTAL 27

Q12 I completed the “Things to Do” assignments before participating in the

Interview Day activities.

Answered: 27 Skipped: 0

Yes

|  |  |  |  |  |  |  |  |  |  |
| --- | --- | --- | --- | --- | --- | --- | --- | --- | --- |
|  | | | | | | |  |  |  |
|  |  |  |  |  |  |  |  |  |  |
|  | | |  |  |  |  |  |  |  |
|  |  |  |  |  |  |  |  |  |  |

No

0% 10% 20% 30% 40% 50% 60% 70% 80% 90% 100%

**ANSWER CHOICES RESPONSES**

Yes 70.37% 19

No 29.63% 8

TOTAL 27

Q13 Approximately, how many miles do you normally live from our campus?

Answered: 27 Skipped: 0

Over 100

75-100

50-75

Less than 50

0% 10% 20% 30% 40% 50% 60% 70% 80% 90% 100%

**ANSWER CHOICES RESPONSES**

Over 100 25.93% 7

75-100 7.41% 2

50-75 18.52% 5

Less than 50

48.15% 13

TOTAL 27

Q14 Were you glad to have had the opportunity to participate in the

Interview Day experience with CUSM-SOM?

Answered: 27 Skipped: 0

Yes

No

0% 10% 20% 30% 40% 50% 60% 70% 80% 90% 100%

**ANSWER CHOICES RESPONSES**

Yes 96.30% 26

No 3.70% 1

TOTAL 27
