# Supplementary material for: The virtues of the virtual medical school interview
Source: Med Educ Online. 2021 Nov 10;26(1):1992820. doi: 10.1080/10872981.2021.1992820 (PMC8592618; doi:10.1080/10872981.2021.1992820)
Supplement: Supplemental Material [file ZMEO_A_1992820_SM8437.zip › Supplementary files/Supplement_5.docx]

**Supplement 5 – Interviewer Questionnaire**

Q1 Overall, which of the following describes your impression of the

Virtual Interview experience as a faculty member?

Answered: 14 Skipped: 0

Outstanding

Excellent

Good

Needs a lot of work

0% 10% 20% 30% 40% 50% 60% 70% 80% 90% 100%

**ANSWER CHOICES RESPONSES**

Outstanding 42.86% 6

Excellent 21.43% 3

Good 35.71% 5

Needs a lot of work

0.00% 0

TOTAL 14

Q2 Overall, I felt the virtual nature of the one-on-one interview with the candidate was as good as if I had experienced the interview in person.

Answered: 14 Skipped: 0

S trongly agree

A gree

Dis agree

S trongly dis agree

0% 10% 20% 30% 40% 50% 60% 70% 80% 90% 100%

**ANSWER CHOICES RESPONSES**

Strongly agree 35.71% 5

Agree 42.86% 6

Disagree 21.43% 3

Strongly disagree

0.00% 0

TOTAL 14

Q4 Did you have any technical issues with Microsoft Teams?

Answered: 14 Skipped: 0

Yes

No

0% 10% 20% 30% 40% 50% 60% 70% 80% 90% 100%

**ANSWER CHOICES RESPONSES**

Yes 28.57% 4

No 71.43% 10

TOTAL 14

Q1 Overall, which of the following describes your impression of the

In-person/on campus experience as a faculty member?

Answered: 10 Skipped: 0

Outstandin

|  |  |  |  |  |  |  |  |  |  |
| --- | --- | --- | --- | --- | --- | --- | --- | --- | --- |
| g | | |  |  |  |  |  |  |  |
|  |  |  |  |  |  |  |  |  |  |
| t | |  |  |  |  |  |  |  |  |
|  |  |  |  |  |  |  |  |  |  |
|  | | | | |  |  |  |  |  |
| f k |  |  |  |  |  |  |  |  |  |

Excellen

Good

Needs a lot o wor

0% 10% 20% 30% 40% 50% 60% 70% 80% 90% 100%

**ANSWER CHOICES RESPONSES**

Outstanding 30.00% 3

Excellent 20.00% 2

Good 50.00% 5

Needs a lot of work

0.00% 0

TOTAL 10

Q2 Overall, I felt the in-person nature of the one-on-one interviews with the candidates were great.

Answered: 10 Skipped: 0

S trongly A gre

|  |  |  |  |  |  |  |  |  |  |
| --- | --- | --- | --- | --- | --- | --- | --- | --- | --- |
| e | | | |  |  |  |  |  |  |
|  |  |  |  |  |  |  |  |  |  |
|  | | | | | |  |  |  |  |
|  |  |  |  |  |  |  |  |  |  |

A gree

Dis agree

0% 10% 20% 30% 40% 50% 60% 70% 80% 90% 100%

**ANSWER CHOICES RESPONSES**

Strongly Agree 40.00% 4

Agree 60.00% 6

Disagree

0.00% 0

TOTAL 10

Q4 As a faculty member did you get to do interviews...

Answered: 10 Skipped: 0

In-person

|  |  |  |  |  |  |  |  |  |  |
| --- | --- | --- | --- | --- | --- | --- | --- | --- | --- |
|  | |  |  |  |  |  |  |  |  |
|  |  |  |  |  |  |  |  |  |  |
|  | | | | | | | |  |  |
|  |  |  |  |  |  |  |  |  |  |

Virtually

Both

(In-person &...

0% 10% 20% 30% 40% 50% 60% 70% 80% 90% 100%

**ANSWER CHOICES RESPONSES**

In-person 0.00% 0

Virtually 20.00% 2

Both (In-person & Virtually)

80.00% 8

TOTAL 10
